# Public Availability of a Genotyped Segregating Population May Foster Marker Assisted Breeding (MAB) and Quantitative Trait Loci (QTL) Discovery: An Example Using Strawberry

**DOI:** 10.3389/fpls.2016.00619

**Published:** 2016-05-09

**Authors:** James F. Hancock, Suneth S. Sooriyapathirana, Nahla V. Bassil, Travis Stegmeir, Lichun Cai, Chad E. Finn, Eric Van de Weg, Cholani K. Weebadde

**Affiliations:** ^1^Department of Horticulture, Michigan State University, East Lansing, MIUSA; ^2^Department of Molecular Biology and Biotechnology, Faculty of Science, University of Peradeniya, PeradeniyaSri Lanka; ^3^United States Department of Agriculture – Agricultural Research Service, National Clonal Germplasm Repository, Corvallis, ORUSA; ^4^Lassen Canyon Nursery Breeding Program, Redding, CAUSA; ^5^United States Department of Agriculture – Agricultural Research Service, Horticultural Crops Research Unit, Corvallis, ORUSA; ^6^Wageningen UR Plant Breeding, WageningenNetherlands; ^7^Department of Plant, Soil and Microbial Sciences, Michigan State University, East Lansing, MIUSA

**Keywords:** *Fragaria × ananassa*, *F. chiloensis*, *F. virginiana*, association mapping, quantitative trait analysis (QTL)

## Abstract

Much of the cost associated with marker discovery for marker assisted breeding (MAB) can be eliminated if a diverse, segregating population is generated, genotyped, and made available to the global breeding community. Herein, we present an example of a hybrid, wild-derived family of the octoploid strawberry that can be used by other breeding programs to economically find and tag useful genes for MAB. A pseudo test cross population between two wild species of *Fragaria virginiana* and *F. chiloensis* (FVC 11) was generated and evaluated for a set of phenotypic traits. A total of 106 individuals in the FVC 11 were genotyped for 29,251 single nucleotide polymorphisms (SNPs) utilizing a commercially available, genome-wide scanning platform (Affymetrix Axiom IStraw90^TW^). The marker trait associations were deduced using TASSEL software. The FVC 11 population segregating for daughters per mother, inflorescence number, inflorescence height, crown production, flower number, fruit size, yield, internal color, soluble solids, fruit firmness, and plant vigor. Coefficients of variations ranged from 10% for fruit firmness to 68% for daughters per mother, indicating an underlying quantitative inheritance for each trait. A total of 2,474 SNPs were found to be polymorphic in FVC 11 and strong marker trait associations were observed for vigor, daughters per mother, yield and fruit weight. These data indicate that FVC 11 can be used as a reference population for quantitative trait loci detection and subsequent MAB across different breeding programs and geographical locations.

## Introduction

The expense of developing a dense linkage map of single sequence repeats (SSRs) (Sargent et al., 2012) and/or single nucleotide polymorphism (SNPs) (Bassil et al., 2015) to conduct marker assisted breeding (MAB) (Collard and Mackill, 2008) is prohibitive for many breeding programs, particularly in the developing countries. Much of this cost can be circumvented; however, if a diverse, segregating population is made available to the global breeding community that has already been genotyped using markers from a published linkage map. That segregating population could be evaluated by the recipient breeding program *in situ* for the traits of interest and then the published linkage map could be used to search for markers associated with traits of interest. The only costs incurred by the recipient breeding program would be those associated with the phenotyping and computer analysis and use of only the relevant markers. The high costs associated with the generation of a dense linkage map of a segregating population could be avoided, saving the recipient breeding program hundreds of thousands of dollars in development costs. Costs could be further reduced by utilizing publicly available association mapping software such as ‘Tassel’ (Bradbury et al., 2007; Khan, 2011).

In this paper, we describe a hybrid population of the octoploid strawberry that should be a rich source of novel genes for the global breeding community. We have genotyped this population for 29,251 SNPs that were previously mapped in another segregating population (Bassil et al., 2015). The objective of this paper is to show how this population can be used by other breeding programs to economically find and tag useful genes for MAB. A similar approach could be used in all crops to make MAB much more available to breeding programs with limited resources.

## Materials and Methods

### Segregating Population

We have generated a hybrid population of the octoploid strawberry consisting of four subspecies. The primary cultivated strawberry, *Fragaria* ×*ananassa* Duchesne ex Rozier, initially arose from a hybridization between *F. chiloensis* (L.) Miller subsp. *chiloensis* forma *chiloensis* and *F. virginiana* Miller subsp. *virginiana* in Europe 250 years ago (Hancock, 1999). Wild-collected clones of both species have been evaluated in multiple locations to identify the possible beneficial traits that could be incorporated into the cultivated strawberry and thereby select elite germplasm (Hancock et al., 2001a,b). Elite selections of *F. virginiana* and *F. chiloensis* were intercrossed in 23 combinations and evaluated in the field in Michigan and Oregon (Hancock et al., 2010). The most impressive family was FVC-11 [(Frederick 9 × LH 50–4) × (Scotts Creek × 2 MAR 1A)], which had the best combination of fruit size, color, and yield and was composed of four different subspecies: *F. virginiana* ssp. *virginiana* from Ontario (Frederick 9, PI 612493), *F. virginiana* ssp. *glauca* from Montana (LH 50-4, PI 612495), *F. chiloensis* ssp. *chiloensis* forma *patagonica* from Chile (2 MAR 1A, PI 602567), and *F. chiloensis* ssp. *pacifica* from California (Scotts Creek, PI 612490). This population likely contains as much diversity as is possible in a single octoploid strawberry family as it is composed of four different subspecies from four distant ecological regions spanning two continents.

### Marker Development

We genotyped 106 individuals in the FVC 11 family utilizing a commercially available, genome-wide scanning platform (Affymetrix Axiom IStraw90^TW^), according to manufactures instructions (Affymetrix, Inc., Santa Clara, CA, USA). This platform was developed as part of the international RosBREED project, focused on enabling marker-assisted breeding through identification and validation of QTLs for traits of importance to breeders (Iezzoni et al., 2010; Bassil et al., 2015). Short-read sequences from one diploid and 19 octoploid accessions were aligned to the diploid *F. vesca* ‘Hawaii 4’ reference genome to identify SNPs and indels for incorporation into the array. A total of 95,062 marker loci (SNPs, indels, and haploSNPs) were included on the array. After removing monomorphic, segregation wise distorted and ambiguous markers by applying necessary data filters in excel, we identified polymorphisms in the FVC 11 family in a subset of 6,594 SNPs that had already been placed in a dense genetic linkage map of the full sib-family ‘Holiday’ × ‘Korona’ (van Dijk et al., 2014; Bassil et al., 2015). This gave us the physical positions of our segregating markers to do a subsequent association analysis.

### Phenotypic Data

Seventy-eight genotypes of the FVC 11 population were evaluated at Benton Harbor, MI in 2007 and 2008 for their daughter plant production, inflorescence number, inflorescence height, crown production, flower number, fruit size, yield, internal color, soluble solids, and fruit firmness. In June 2007, two to three replicates (runner plants) of each genotype were planted in the field in Benton Harbor, MI, in a randomized complete block design. Plants were set in rows at 1.2 m × 1.2 m spacing and all runners were trained by cross-cultivation into a 0.5 m wide square.

A total of three random inflorescences were selected per mother and daughter plant and their heights were measured from crown to tip and their flower numbers were counted. The number of crowns was also counted on each mother plant and the three daughter plants as well as the total number of daughters produced by each mother plant (original plants set in field). Overall plant vigor was estimated on a 1–7 (least to most vigorous) scale based on plot fill and individual plant vigor. The first five ripe fruit were harvested to determine an average fruit weight, and after another five fruits had ripened all ripe and unripe fruits were picked to determine yield. Fruit firmness (g mm^-2^) was measured on five ripe fruit per plot (when available) using the compression test of BioWorks’ FirmTech 2 (Wamego, KS, USA). Two ripe fruits from each replication were cut in half and percent internal color was estimated visually based on how deep the color penetrated the flesh. Soluble solids were taken by squeezing one drop of juice onto the handheld refractometer from the two fruits for two separate readings. These data were previously reported by Stegmeir et al. (2010).

### Association Analysis

Marker-trait associations were determined by using TASSEL 5 software (Bradbury et al., 2007). Diallelic SNP markers were called for genotypes in which homozygous classes were designated as 0 or 1 and heterozygous class was designated as 0.5. The monomorphic markers, the markers with ≥15% missing data and markers whose genotypes were genetically ambiguous were removed prior to the analysis. The numerical marker and trait data were uploaded to Tassel and kinship was calculated as in a classical association analysis. Then MLM mapping was used to find significant marker trait associations. The probability (p) estimate of [-log10(p)] was used to represent the strength of the associations and the threshold was set to 3.00 for reporting most significant marker-trait associations.

## Results and Discussion

### Phenotypic Variability

As was previously reported by Stegmeir et al. (2010) significant variation was observed among the progeny for their inflorescence number, inflorescence height, crown production, flower number, fruit size, yield, internal color, soluble solids, fruit firmness, and plant vigor (**Table 1**). Coefficients of Variations (CVs) ranged from 10% for fruit firmness to 68% for yield. When progeny means were compared with those of the parental means, many traits exhibited transgressive segregation, most notably yield, and fruit weight. Of the two parents, the *F. virginiana* had the highest value for vigor, crown production, inflorescence number, inflorescence height, flower numbers, yield, and depth of fruit color, while the *F. chiloensis* had the highest values for fruit weight and firmness.

**Table 1:** The mean, range, and coefficient of variation (CV) for 15 traits evaluated in the strawberry FVC 11 family at Benton Harbor, MI in 2008 (Stegmeir et al., 2010).

Trait	Means of parents		FVC -11 family		
	Fragaria *chiloensis*	*F. virginiana*	Mean	Range	CV (%)
Plant vigor	3.0	5.7	3.9	1.5–6.0	27
Daughters per mother	10.0	10.7	8.3	0.0–20.0	58
Crowns per mother	1.7	3.3	4.6	1.0–11.3	48
Crowns per daughter	1.0	2.0	1.4	1.0–3.0	21
Inflorescences per mother	1.7	5.7	6.8	1.0–16.3	54
Inflorescences. per daughter	1.2	2.6	1.7	0.0–4.1	29
Inflorescences height (cm)	7.0	14.8	9.7	4.1–17.7	27
Flowers per infl.	3.7	6.6	5.5	3.5–8.8	20
Fruit wt. (g)	3.1	1.9	6.1	1.0–12.6	41
Yield per plant (g)	1.0	2.0	9.0	0.6–33.6	68
Total yield (g)	33.3	68.6	213.7	22.9–651.0	68
Soluble solids	11.1	10.9	8.4	5.1–11.0	14
Fruit firmness	157.0	141.1	153.2	120.9–192.2	10
Internal color (%)	17.5	62.9	54.2	32.5–80.0	19

### Genotypic Variability and Association Analysis

The FVC 11 segregating population proved to be highly polymorphic. Out of the 6,594 SNPs that had been placed on the genetic linkage map of ‘Holiday’ × ‘Korona’ by Bassil et al. (2015), we found 37.42% to be polymorphic in our FVC-11 population. In the subsequent QTL analysis, we found a number of SNP markers that were closely linked to important horticultural traits, with highly significant *p* values and -log10 (*p*) values of ≥3.0. We discovered a SNP on LG 6 (AX-89796183) that was associated with both plant vigor and the production of daughter plants. SNPs in two regions of LG 2 were associated with fruit weight (AX-89904664; AX-89880679, and AX-89823518). A SNP on LG 5 (AX-89849271) was associated with yield per plant, while two other SNPs on LG 1 (AX-898760359) and LG 6 (AX-89898803) were significantly associated with yield per plot (**Table 2**).

**Table 2:** Significant marker-trait associations found in the FVC 11 population.

Trait	Marker	Chromosome	Position	*p*	[–log10(p)]
Vigor	AX-89796183	6	1865527	0.000496	3.30
Daughters per mother	AX-89796183	6	1865527	0.000827	3.08
Yield per plant (g)	AX-89849271	5	26321147	0.000441	3.36
Total yield (g)	AX-89876035	1	7946370	0.000745	3.13
Total yield (g)	AX-89898803	6	35968025	0.000611	3.21
Fruit wt. (g)	AX-89904664	2	9843555	0.001058	2.98
Fruit wt. (g)	AX-89880679	2	9923170	0.001058	2.98
Fruit wt. (g)	AX-89823518	2	9927708	0.000231	3.64

## Conclusion

Based on the results described above, it is clear that the FVC-11 population is highly polymorphic for numerous horticulturally important traits and could provide useful genetic variability to other strawberry breeding programs. It is likely that the population also segregates for a number of other important horticultural traits that we did not evaluate. The original *F. virginiana* parent Frederick 9 is resistant to powdery mildew and leaf scorch, and very highly resistant to the northern root knot nematode. The other original *F. virginiana* parent LH-50-4 is unusually cold-hardy and is also very highly resistant to the northern root knot nematode. The *F. chiloensis* parent Scotts Creek has high salt and drought tolerance, and low nutrient needs. The other *F. chiloensis* parent MAR 1A also has high salt and drought tolerance, and low nutrient requirements. The environmental adaptations of the parents ranged from the Mediterranean climates where Scotts Creek and MAR 1A grew, to the high elevation Rocky Mountain habitat of LH 50-4 and the mid-south Canadian, continental climate of Frederick 9.

We have demonstrated how an existing, soon to be published linkage map can be used to find QTL cheaply, using publically available software (e.g.,: ‘TASSEL’) (Bradbury et al., 2007; Khan, 2011) and a commercially available SNP array. We will maintain the FVC 11 family at Michigan State University and will make plants available to other interested strawberry breeders until at least December 31, 2017, so that other global programs can search for breeder-friendly markers and use these informative markers themselves. We ask only that the recipients pay for any required phytosanitary analysis and shipping costs.

## Author Contributions

JH – Designed study and was primary source of funding, wrote manuscript. SS – Collected leaf samples for SNP analysis, conducted QTL analysis, generated tables for manuscript NB – Supervised DNA extraction and development of SNPs. TS – Collected phenotypic data. LC – Organized SNP data for analysis. CF – Generated FVC family that was analyzed. EW – Provided unpublished linkage map for QTL analysis. CW – Was secondary source of funding, played integral role in planning, and interpretation of data.

## Conflict of Interest Statement

The authors declare that the research was conducted in the absence of any commercial or financial relationships that could be construed as a potential conflict of interest.
